# Supplementary material for: Chromothripsis during telomere crisis is independent of NHEJ, and consistent with a replicative origin
Source: Genome Res. 2019 May;29(5):737–49. doi: 10.1101/gr.240705.118 (PMC6499312; doi:10.1101/gr.240705.118)
Supplement: Supplemental Material [file supp_gr.240705.118_Supplemental_file_1.zip › contigs/annotated_contigs/DB57/contig.2.DB57_length_400_mean_cov_5.4675.docx]

**DB57_length_400_mean_cov_5.4675**

```
TCCCCTCTAACAAAATATACTCTTCCTTTATCCTTTATTTAGATATTAAATCACAGCCTTTTATTATGAAACCATTTTATAAATTAAAA
 >chr1:174536546-174536658 - E=6e-56
TATAAGGCATGAGATAAATGGCT|T|AAACATGTTTTTAAAATTAGTTTGGTGTGGTGGTGCATGCCTATAGTCCTAGCTACTTGAGAG
                         >chr1:174704021-174704283 - E=1e-127
GCTGAGGCAGGAGGATTGCTGGAGCCCAGGAATTTGAGGCGGCAGTAAGCTATGATTGTGCCACTGTACTCCAGCCTGGGCAACAAAGT

GAGACCCCATATCTTAAAAAAAAAAAAAAGAGAGAGATCCTAATTTCCCAAATTAAATGAGCATACTTCCCACTTAAAAAAAAAACTAA

ATAAGCATAAG|ATTTACTGTATGTAAAAAGAACAAATAAAAATGAT
```
